# Challenges past, present and future for *Endocrine Connections*

**DOI:** 10.1530/EC-23-0436

**Published:** 2023-11-10

**Authors:** Adrian J L Clark

**Affiliations:** 1 Editor-in-Chief, *Endocrine Connections*

As I come to the end of my term as editor-in-chief of *Endocrine Connections,* it is useful to reflect on the developments in the journal and in scholarly publishing in general over recent years. I am naturally happy that* Endocrine Connections* has maintained its place as one of the leading fully open-access endocrine journals covering a broad range of clinical and basic endocrine science whilst at the same time supporting its two parent societies – the *Society for Endocrinology* and the *European Society of Endocrinology*. Article download metrics now match those of the leading specialist endocrine journals, whilst we maintain some of the lowest article publication charges in the field. These achievements are supported by a highly engaged and dedicated board of senior editors drawn from endocrinologists across the globe and I am greatly indebted to them for their support, advice and hard work.

My first role as an editor-in-chief was for the *Journal of Endocrinology* which I took on 15 years ago. It is interesting to observe how the challenges in endocrine publishing have changed over this time. At that time a major concern was in detecting and dealing with fraudulent data and in distinguishing inadvertent from intentional plagiarism. Although these problems have not gone away, I believe them to be less prevalent – with one major exception. That exception has been the rise of the papermill. We, and others, have written about this previously (1, 2), arguing that such massive fraud reflects badly on the excellent science that papermill-hosting countries can and do produce. It does seem that this problem has receded over the last year, at least in this journal, although the other ominous possibility remains that papermills have become so sophisticated that they avoid detection.

A new phenomenon is the rise of artificial intelligence (AI) as exemplified by applications such as ChatGPT. Clearly, AI can be of great assistance in supporting the quality and competence of scientific reports, and my view is that this is acceptable in scientific publication – although authors should be obliged to declare the use of this technology in their paper. This area will undoubtedly develop over the coming years.

The greatest challenge that faces scientific publication today though is peer review. Good-quality review is fundamental to scientific advancement, but if we consider the numbers one has to question whether this is sustainable in its current form. In 2022, for example, there were just under 21,000 papers published in the Diabetes and Endocrinology category of the Science Citation Index. If each of these had two peer reviews, with each requiring perhaps 1.5 h of individual time (including revisions), around 30 years of academic time would be required just to cope with the published output in 2022. Of course, many of these papers will be reviewed by two or more journals before being accepted, and many will never be published, so perhaps between 70 and 100 years of expert academic time is a more realistic estimate. This would roughly equate to a cost of around 10 million euro/US dollars/GB pounds per year to peer review in Diabetes and Endocrinology alone. Currently, this service is provided at no cost. I find it hard to imagine that academic or health-care provider employers will continue to sustain this situation when the financial pressures on their organisations are ever-mounting, and there is certainly evidence now of universities discouraging academics from providing this function. Arguments that, as scientists, we have a moral obligation to conduct peer review, or that it is undoubtedly beneficial to the reviewer’s practical and intellectual development, have little traction when such financial restraints come into play.

In keeping with this, this journal is undoubtedly not alone in finding it increasingly hard to recruit good-quality reviewers in a timely fashion, and, in my view this is the greatest challenge for the future of scholarly publication. In the case of *Endocrine Connections* we have developed a dedicated and trained Reviewer Editorial Board who often provide vital assistance in reviews, but this cannot be a long-term solution.

Could it be that AI might substitute for expert peer review? Currently it seems that AI can ‘review’ the language and readability of a paper and perhaps contribute to the best use of citations or the appropriateness of scientific and statistical methods. This topic has been reviewed in detail (e.g. Checco *et al.* (3)), but the ability to critically understand a hypothesis, the practicalities of testing that hypothesis and then to critique the author’s interpretation of the findings is, I believe, not presently possible, and whilst it may become feasible, given the speed with which we have seen AI tools develop in recent years, I am doubtful that the scientific community would ever accept science reviewed this way.

If AI is not the solution, it seems to me that publishers will have to pay reviewers – with these costs obviously being passed on to authors at the time of manuscript submission. Payment of an honorarium to peer reviewers is already accepted in the review of research funding, and it seems to me inevitable that this will come to scholarly publishing soon. Whether this will have an inhibitory effect on the quantities of articles submitted and any tendency to submit to higher impact journals in the first instance remains to be seen – but this may not be a bad thing.

So, it is with great sadness that I step down as editor-in-chief, knowing that the journal will remain in the very competent hands of Professor Faisal Ahmed who will take over from me and who will have to address some of these interesting challenges in the coming years. I wish him and the journal every success.

## Declaration of interest

The author declares that there is no conflict of interest that could be perceived as prejudicing the impartiality of this editorial.

## Funding

This work did not receive any specific grant from any funding agency in the public, commercial or not-for-profit sector.
